# Cell-Free Co-Translational Approaches for Producing Mammalian Receptors: Expanding the Cell-Free Expression Toolbox Using Nanolipoproteins

**DOI:** 10.3389/fphar.2019.00744

**Published:** 2019-07-03

**Authors:** Megan L. Shelby, Wei He, Amanda T. Dang, Tonya L. Kuhl, Matthew A. Coleman

**Affiliations:** ^1^Lawrence Livermore National Laboratory, Livermore, CA, United States; ^2^University of California at Davis, Davis, CA, United States

**Keywords:** cell-free, co-translation, nanolipoprotein particle, nanodisc, membrane proteins

## Abstract

Membranes proteins make up more than 60% of current drug targets and account for approximately 30% or more of the cellular proteome. Access to this important class of proteins has been difficult due to their inherent insolubility and tendency to aggregate in aqueous solutions. Understanding membrane protein structure and function demands novel means of membrane protein production that preserve both their native conformational state as well as function. Over the last decade, cell-free expression systems have emerged as an important complement to cell-based expression of membrane proteins due to their simple and customizable experimental parameters. One approach to overcome the solubility and stability limitations of purified membrane proteins is to support them in stable, native-like states within nanolipoprotein particles (NLPs), aka nanodiscs. This has become common practice to facilitate biochemical and biophysical characterization of proteins of interest. NLP technology can be easily coupled with cell-free systems to achieve functional membrane protein production for this purpose. Our approach involves utilizing cell-free expression systems in the presence of NLPs or using co-translation techniques to perform one-pot expression and self-assembly of membrane protein/NLP complexes. We describe how cell-free reactions can be modified to render control over nanoparticle size and monodispersity in support of membrane protein production. These modifications have been exploited to facilitate co-expression of full-length functional membrane proteins such as G-protein-coupled receptors (GPCRs) and receptor tyrosine kinases (RTKs). In particular, we summarize the state of the art in NLP-assisted cell-free coexpression of these important classes of membrane proteins as well as evaluate the advances in and prospects for this technology that will drive drug discovery against these targets. We conclude with a prospective on the use of NLPs to produce as well as deliver functional mammalian membrane-bound proteins for a range of applications.

## Introduction

Biochemical and structural characterization of a transmembrane protein requires isolation of the purified molecule independent of the many protein neighbors that are contained in the same membrane. This can be accomplished using detergents that are capable of dissociating and solubilizing the individual membrane protein components by acting as a micelle-forming lipid bilayer mimetic, then purifying the protein of interest using conventional techniques in the presence of a detergent (Garavito and Ferguson-Miller, 2001). Although incorporation of the protein of interest into a mixed lipid/detergent micellar phase is an effective solubilization strategy, there are several downsides to this approach if the goal is the structural and functional assessment of the native membrane protein. Exchange between protein-bound lipid and detergent within micelles can lead to the gradual co-concentration of detergent with the protein during purification. Loss of functionally important bound phospholipids can lead to the deterioration of activity and may perturb protein conformation to the point of instability and aggregation. These factors contribute to the reputation of purified membrane proteins as unstable and temperamental, but there is increasing recognition that retaining protein function and stability while enabling adequate solubilization and purification may be achieved through improved membrane mimetics.

These problems are commonly addressed by reconstitution procedures that include the addition of lipids during detergent removal, resulting in spontaneous formation of lipid bilayer vesicles into which the protein is incorporated. Reconstitution by these methods can be effective in maintaining the solubilized protein of interest in a near-native lipid environment, but has two important limitations: (Garavito and Ferguson-Miller, 2001) the purified proteins molecules are now part of much larger entities, proteoliposomes, which are both heterogeneous in size and sparingly soluble and thus cannot be manipulated or analyzed by many of the techniques applied to soluble purified proteins; and (Bowie, 2001) the protein is embedded in an extended two-dimensional solution of lipid bilayer in which multiple protein molecules incorporated into the same bilayer can readily interact or oligomerize unpredictably and uncontrollably.

**Figure 1:**
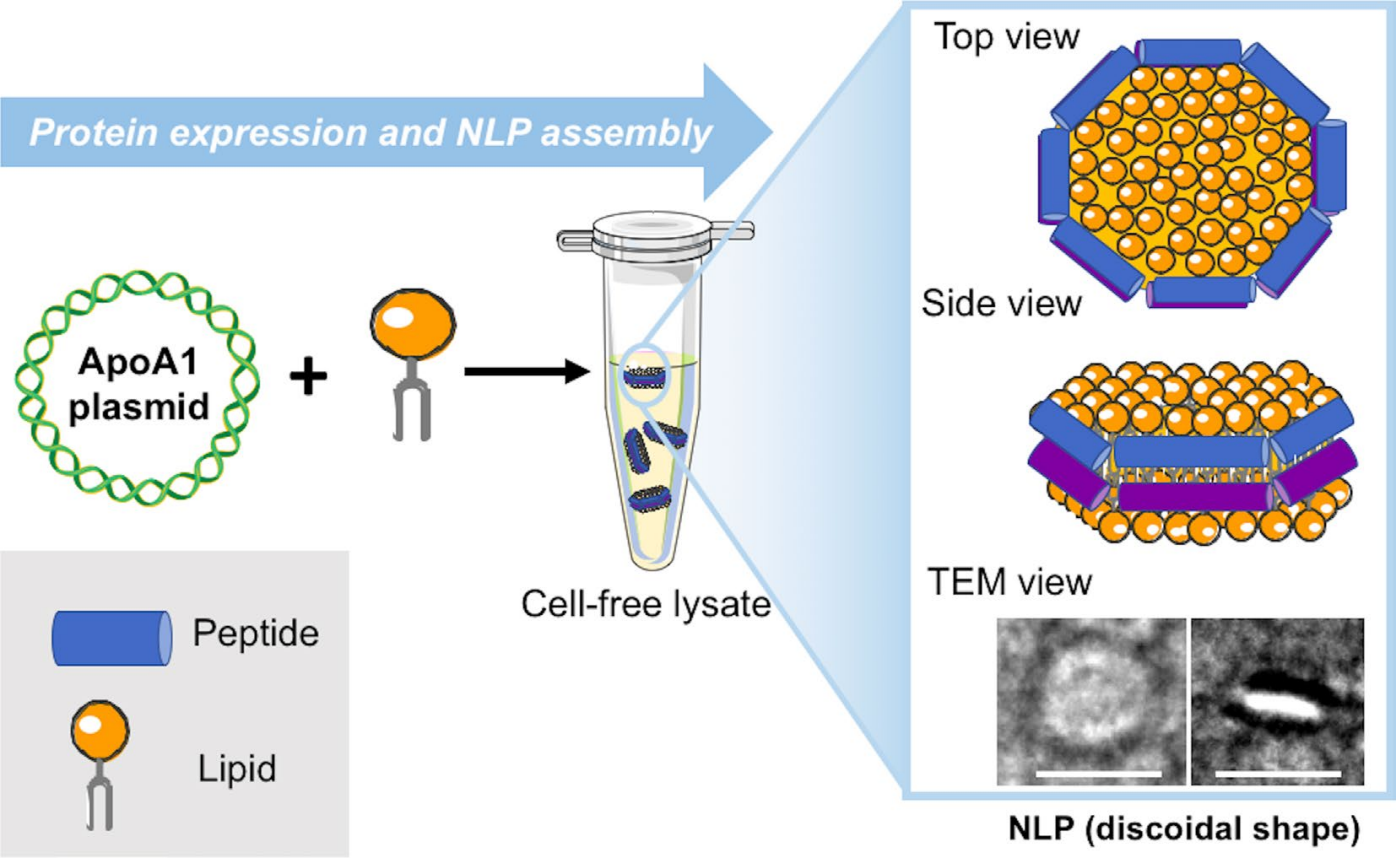
Schematic for cell-free production of self-assembled nanolipoprotein particles (NLPs). Constituents (DNA, lipids, and cell-free extracts) are combined in a single reaction vial. The cell-free lysates utilize T7-coupled transcription and translation to produce fully formed disc shaped nanoparticles as imaged with transmission electron microscopy (TEM) (inset). The white scale bars are 20 nm.

**Figure 2:**
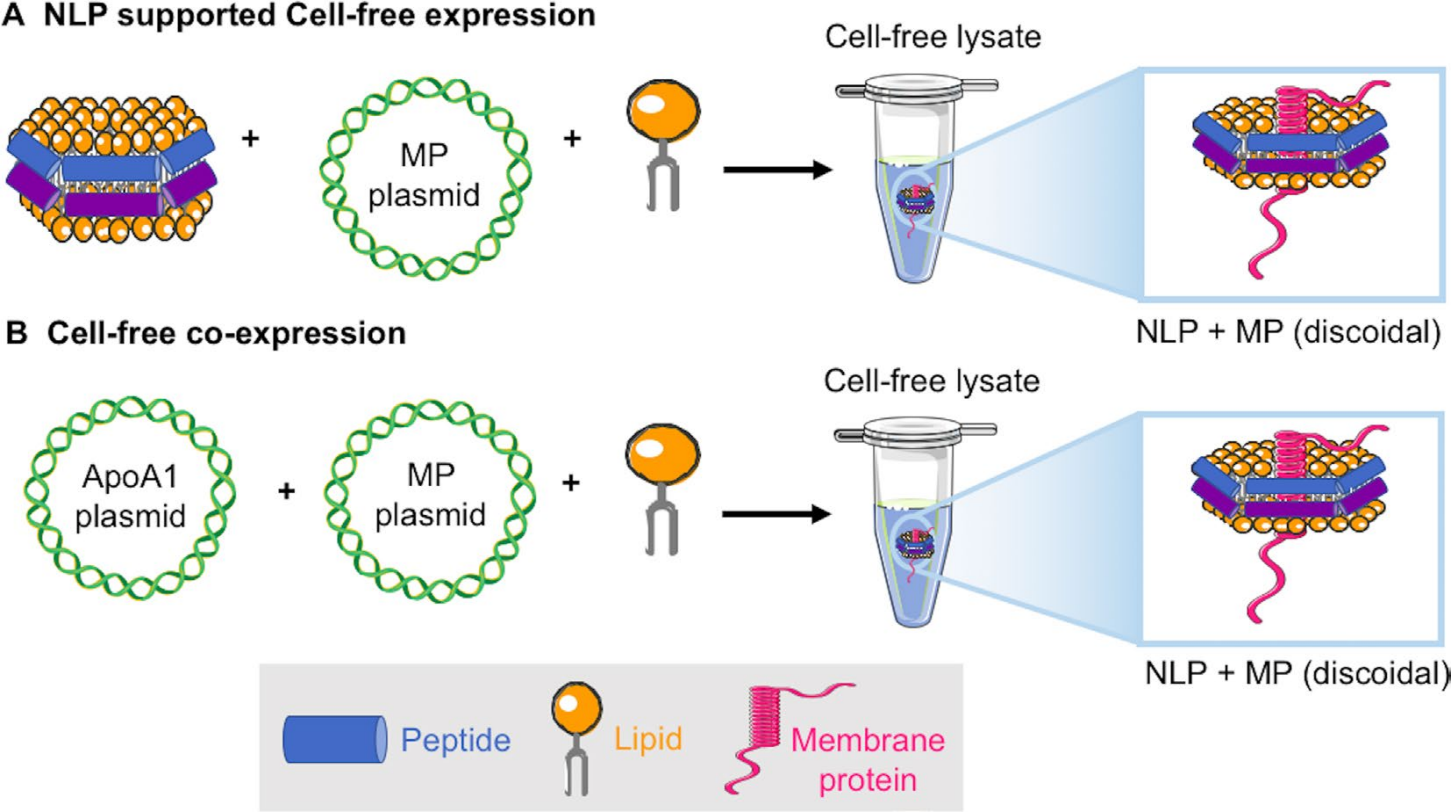
Schematic of two approaches for cell-free production of membrane proteins solubilized in NLPs: **(A)** The addition of fully formed NLPs, DNA encoding the membrane protein of interest, and additional lipid to a cell-free extract results in NLP solubilized receptor (Katzen et al., 2008). **(B)** Co-translation uses the addition of only plasmids encoding the ApoA1 and membrane protein of interest and lipid to a cell-free extract resulting in NLP solubilized receptor (Cappuccio et al., 2008).

Over the last decade, multiple new techniques beyond the standard use of lipids and detergents have become available to address the challenges related to these approaches. These include amphipol solubilization (Tribet et al., 1996; Bowie, 2001), insertion into bilayer-containing nanostructures, such as nanolipoprotein particles (NLPs) (or “nanodiscs”) (Ritchie et al., 2009; Schuler et al., 2013) and association with styrene–maleic acid lipid particles (SMALPs) (Knowles et al., 2009). We have primarily focused on the development and application of a single-step cell-free co-expression method that results in subsequent self-assembly of NLPs. The NLPs assemble during the cell-free reaction when apolipoprotein produced from a plasmid provides a supporting scaffold that mixes with a population of phospholipids to form a protein encapsulated disc of lipid bilayer in an aqueous environment (**Figure 1**) (Jonas, 1986; Bayburt et al., 2002). This finding is striking because the nanoparticles can rapidly assemble as homogeneous entities within a simple cell-free reaction without the need to alter the lipid to protein ratio, select detergents to solubilize and stabilize proteins, or for extensive purification steps. All or some of these experimental parameters commonly require optimization using traditional assembly approaches. The output of the one-pot reaction was quickly adapted for producing and solubilizing functional membrane proteins supported in NLPs as outlined in **Figure 2**. This unique combination of cell-free expression and near-simultaneous NLP assembly offers a significant improvement over solubilization with proteoliposomes and detergents, which require multiple steps over a lengthy period to obtain the membrane protein of interest. The cell-free production of NLPs provides a bilayer mimic that closely resembles the cell membrane, allowing membrane proteins to retain function while supported in the nanoparticle (Bayburt et al., 2006; Leitz et al., 2006). NLPs have distinct advantages over currently used proteoliposomes in terms of particle size monodispersity and batch consistency: the presence of the circular protein belt constrains the dimensions of the bilayer and ensures narrow NLP particle size distributions with little variation between preparations (Chromy et al., 2007). Several physical characterization techniques, including size-exclusion chromatography, dynamic light scattering, and electron microscopy demonstrate that “empty” NLPs (with no membrane protein bound) are monodisperse with size and shape consistent with a discoidal lipid bilayer (Blanchette et al., 2009a). The constraint of the protein belt scaffold also makes NLPs relatively stable over time compared with detergent micelles. Although NLP synthesis and physical characterization have been rigorously developed over the last decade, there has been growing interest in functional protein incorporation into the discs using the one-pot approach (Cappuccio et al., 2008; Katzen et al., 2008; Baker et al., 2009; Cappuccio et al., 2009).

A variety of membrane proteins have been incorporated into NLPs using traditional assembly methods, which have required detergent removal during the assembly process. Examples using traditional assembly include rhodopsins, G-protein-coupled receptors (GPCRs), cytochrome P450, transporters, functional channels, and other assorted receptors (Klammt et al., 2007; Whorton et al., 2007; Kobashigawa et al., 2011; Yang et al., 2011). We have primarily focused on taking advantage of cell-free methods in the absence of detergents to rapidly produce a variety of membrane proteins embedded in NLPs that are functionally and structurally active after *de novo* generation. We have focused on two cell-free methods, cell-free generation of the encapsulated membrane protein using pre-formed NLPs (Katzen et al., 2008) (**Figure 2A**), and co-translation of apolipoprotein and the membrane protein in the presence of lipid resulting in *de novo* assembly of the membrane protein supported by the NLP (Cappuccio et al., 2008) (**Figure 2B**). The co-translation or “single-pot” method also has the advantage that expression levels of protein are not limited by the input of preformed NLPs and multiple-protein complexes can be formed within the single reaction (Cappuccio et al., 2008; Coleman et al., 2016).

The “single-pot” method has become the primary technique we employ for generating and characterizing proteins of interest. As shown in **Table 1**, not only are individual membrane proteins solubilized by this technique but also functional oligomeric complexes can be recapitulated within the nanodisc, and they are amenable to multiple biophysical characterization tools (Cappuccio et al., 2008; Coleman et al., 2016). The rapid turn-around time of the cell-free system also allows for quick screening, scaling, and modification of conditions used for expression. Co-translation requires plasmid input for the apolipoprotein along with the plasmid encoding the membrane protein of interest, both of which require compatible promoters for ideal expression. The ratio of the two plasmids encoding the protein of interest and the apolipoprotein can be screened based on plasmid ratios to maximize expression levels for the protein of interest. Normally, this screen can be easily accomplished in a single day. For example, small-scale reactions are first run at 25 to 50 µL to test and optimize plasmid input levels (1–4 h). The best conditions can be selected based on highest expression levels or best solubility can then be scaled to 0.1 to 1 ml (4 h—overnight) (Cappuccio et al., 2008). Reactions are also supplemented with phospholipids for membrane bilayer formation in the resulting nanoparticles. Most cell-free reactions are robust and can normally accept an excess of 25% volume, which we take advantage of by adding lipids at varying concentrations (20–68 mg/ml). As described, one-pot cell-free expression and self-assembly of NLPs avoids many of the steps and pitfalls of membrane protein expression, purification, and detergent solubilization, which are still necessary for conventional nanodisc assembly (Jonas, 1986; Bayburt et al., 2002).

**Table 1:** Examples of cell-free co-translated membrane proteins associated with nanolipoprotein particles (NLPs).

Membrane protein	Protein molecular weight	NLP diameter (nm)	Measuring technique	Cell-free references
Yop B + Yop D	42 + 33 kDa	18.9 ± 4.9	AFM	Coleman et al., 2016
NK1R	46 kDa	10.3 ± 7.5	FCS	Gao et al., 2012
β2AR	47 kDa	33.0 ± 3.0	TEM	Patriarchi et al., 2018
BR	28 kDa	7.8 ± 2.8	FCS	Gao et al., 2011
MOMP	40 kDa	39.8 ± 4.2	DLS	He et al., 2017
ERBB2 (HER2)	138 kDa	12.5	SAXS	He et al., 2015
EGFR	134 kDa	30.1	DLS	Quinn et al., 2019

Yop, Yersinia outer protein; NK1R, neurokinin1 receptor; β2AR, beta-2 adrenergic receptor; BR, bacteriorhodopsin; MOMP, chlamydia major outer membrane protein; AFM, atomic force microscopy; FCS, fluorescence correlation spectroscopy; TEM, transmission electron microscopy; DLS, dynamic light scattering; SAXS, small-angle X-ray scattering.

Below, we further detail the role cell-free co-expression has played in characterizing full-length functional membrane protein mammalian receptors, such as GPCRs and receptor tyrosine kinases (RTKs). In particular, we summarize the state of the art in NLP-assisted generation of these two important classes of membrane proteins as well as evaluate the advances in and prospects for this technology that will drive future studies.

## Cell-Free NLP Co-Expression for Characterizing GPCRs

GPCRs are one of the largest families of membrane-bound cell-surface receptors involved in modulating signal transduction. This class of receptors plays an essential role for a broad range of physiological responses that transduce extracellular signals (e.g., photons, odorants, hormones, nucleotides, nucleosides, peptides, lipids, and proteins) into intracellular responses through coupling with G proteins (Lundstrom, 2005; Lundstrom, 2006). Approximately 30% to 40% of all drugs are targeted to GPCR activity (Hanson and Stevens, 2009). To obtain pure and soluble GPCRs, while maintaining proper folding and a native conformational landscape, requires individual receptor molecules to be in a native-like environment. Currently, cell-based expression and purification is the primary method of GPCR production, which is time-consuming and labor intensive (Milic and Veprintsev, 2015; Saarenpaa et al., 2015). Cell-free synthesis of membrane proteins has emerged as a great alternative to cell-based methods, and aids functional characterization of these receptors (Klammt et al., 2007). Importantly, many different types of lysates, such as insect and mammalian derived cell-free lysates, can be used to make GPCRs (Sonnabend et al., 2017; Zemella et al., 2017) and may aide in post-translational modification, such as complex glycosylation.

**Figure 3:**
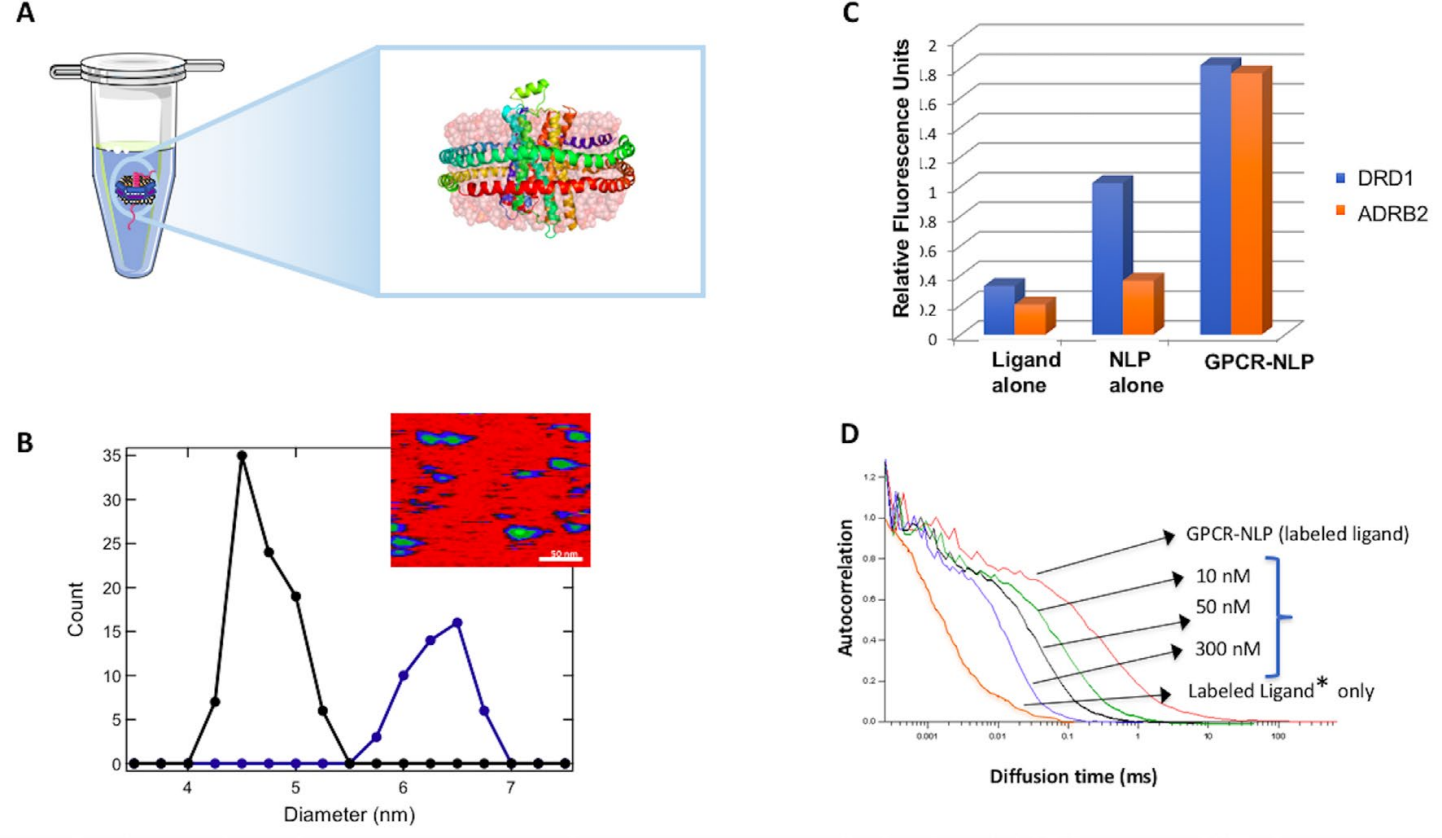
Cell-free co-expression for production of G-protein-coupled receptor (GPCR)-NLP complexes. **(A)** Illustrated β_2_-adrenergic receptor (β_2_AR) embedded in an NLP *via* cell-free expression and self-assembly. **(B)** Atomic force microscopy (AFM) image of NLPs with inserted GPCRs to determine the insertion rate based on the increase in the diameter of the disc. The inset shows an AFM image. The scale bar is 50 nm. **(C)** A histogram showing ligand binding for cell-free produced dopamine receptor D1 (DRD1) and β_2_AR (ADRB2). Data is based on fluorescent filter binding. **(D)** Fluorescence correlation spectroscopy (FCS) analysis of ligand concentration dependent binding is illustrated for the NK1 GPCR. An increase in the ligand concentration shows increased binding based on the shift of the spectrum to the right.

This important group of proteins has also been assembled in nanodiscs prepared using traditional approaches. The β_2_-adrenergic receptor (β_2_AR) (**Figure 3A**) and rhodopsin GPCRs have been reconstituted previously in nanodiscs using detergent-based methods (Bayburt et al., 2007; Sunahara et al., 2007) and shown to efficiently activate the associated G protein (transducin for rhodopsin) in functional assays. In these studies, agonist binding to NLP-reconstituted GPCRs were found to be more consistent with cell-membrane assays than those within detergent micelles or lipid vesicles, which supported the use of nanoparticle-based production for further structural studies. One potential benefit of using the NLPs/nanodisc versus liposome or detergent micelle systems is the fact that orientation of the membrane-bound protein did not limit access to ligand binding sites. Preparations, such as microsomes, have also been highly successful for preparing small yields of functional GPCR proteins. However, these native lipid-based systems are not soluble and result in very heterogeneous preparations.

The use of NLPs offers a native-like environment for maintaining the structure and functionality of these receptors (Leitz et al., 2006; Denisov and Sligar, 2016), whereas the single-pot method enables rapid and scalable production. This combined approach is both simple and elegant for GPCR production (**Figure 3**) (Gao et al., 2012; He et al., 2015). We have used this strategy to successfully express a number of GPCRs including the model GPCR-like protein bacteriorhodopsin (bR), human neurokinin (NK1R), human beta-adrenergic (β _2_AR) and human dopamine (DRD1) receptors with no amino acid sequence alteration while maintaining their biological function (Gao et al., 2011; Gao et al., 2012). On average we can produce 100 µg/ml of GPCRs with a 35% insertion rate (% of NLPs that contain GPCRs) of the embedded membrane protein based on Atomic Force Microscopy measurements (**Figure 3B**). These GPCR-NLPs are capable of interacting with their respective ligands as demonstrated by a fluorescent-ligand filter binding assay and by fluorescent correlation spectroscopy (FCS) (**Figure 3C and D**). In these studies, the receptors are all free of tags and protein fusions. We normally limit tags and fusion partners to NLP belt proteins so that the receptors retain a native conformation. Using cell-free systems to express functional, stable β_2_AR in the presence of detergent solubilized lipid assembled NLPs, the authors required fusion constructs containing a T4 lysozyme within NLPs to stabilize the protein (Yang et al., 2011). This same T4 lysozyme fusion has also proven necessary for obtaining many of the current GPCR crystal structures (Stauch and Cherezov, 2018). The majority of our studies have instead focused on unaltered full-length receptors for functional characterization.

Traditionally, GPCRs are characterized as functional through ligand binding. Ideally, as GPCR signaling involves many complex conformational changes that initiate downstream G-protein signaling cascades, a full functional characterization of *in vitro*-synthesized GPCRs should include a demonstration of the downstream signaling events. This is of particular interest given that NLPs have been used primarily for biochemical characterization. In our recent study where a full length β_2_AR was produced utilizing *E. coli* cell-free lysates, the activity of the receptor was demonstrated through the activation of G-protein upon ligand binding both *in vitro* and in mammalian cells. This showed that the NLP association allowed for cellular uptake of cell-free–produced β_2_AR, which in turn triggered G-coupled signal transduction and downstream cyclic adenosine monophosphate production. Furthermore, β_2_AR produced *in vitro* was able to rescue the phenotype in a knockout cell line in an *ex vivo* wound-healing model (Patriarchi et al., 2018). This was the first demonstration of applying cell-free technology for receptor replacement and/or augmentation in living cells.

## Cell-Free NLP Co-Expression Systems for Characterizing Function for the ERBB Family of Receptor Tyrosine Kinases (ERBB2 and EGFR)

The ERBB (erythroblastic oncogene B) family of RTKs are membrane-bound proteins that participate in tumor proliferation when overexpressed or showing increased activity above normal cellular levels (Moasser, 2007). For example, a member of the ERBB family ERBB2, also known as HER2, is amplified in about 30% of all breast cancers and overexpression of EGFR (epidermal growth factor receptor) is found in more than 50% of non-small cell lung cancer (Normanno et al., 2006; da Cunha Santos et al., 2011). The ERBB family of receptors are membrane proteins with single transmembrane helices. Like most membrane proteins, the crystal structure of HER2 has been limited to exclusively the intracellular or extracellular soluble domains (Cho et al., 2003) but the structure of the full-length receptor has yet to be resolved. Historically, the ERBB family of proteins are drug targets for cancer treatment because they are implicated in tumor proliferation. Genentech’s trastuzumab (Herceptin) is one of the first antibody drugs for targeted therapy of breast cancer and targets the small ERBB2 extracellular domain (Carter et al., 1992). Unfortunately, patients often develop resistance to trastuzumab treatment and many patients that display overexpression of HER2 do not respond to the drug (Tai et al., 2010; Phillips et al., 2014). To understand the mechanism of such resistance, we need to have a better understanding of the structure of ERBB2. This would necessitate producing a full-length and structurally homogeneous preparation of the receptor.

Traditionally, ERBB proteins are purified from mammalian cell membrane extracts with the help of detergents (Penuel et al., 2002). It is increasingly evident that the cell membrane environment plays a critical role in maintaining native conformation and, therefore, function for membrane proteins in general, making a strong case for development of a membrane mimetic-based solubilization strategy for ERBB. A bigger challenge is that many other cellular components that associate with ERBB receptors, for example other ERBB family member proteins, are often co-purified with the target protein. Cell-based and insect cell expression systems combined with NLPs have been used to isolate ERBB receptors (Mi et al., 2008; Scharadin et al., 2017). However, overexpression relied on fusion tags and detergent solubilization. Such alterations and impurities (even at low concentration) may have profound impact on the function of the receptor.

**Figure 4:**
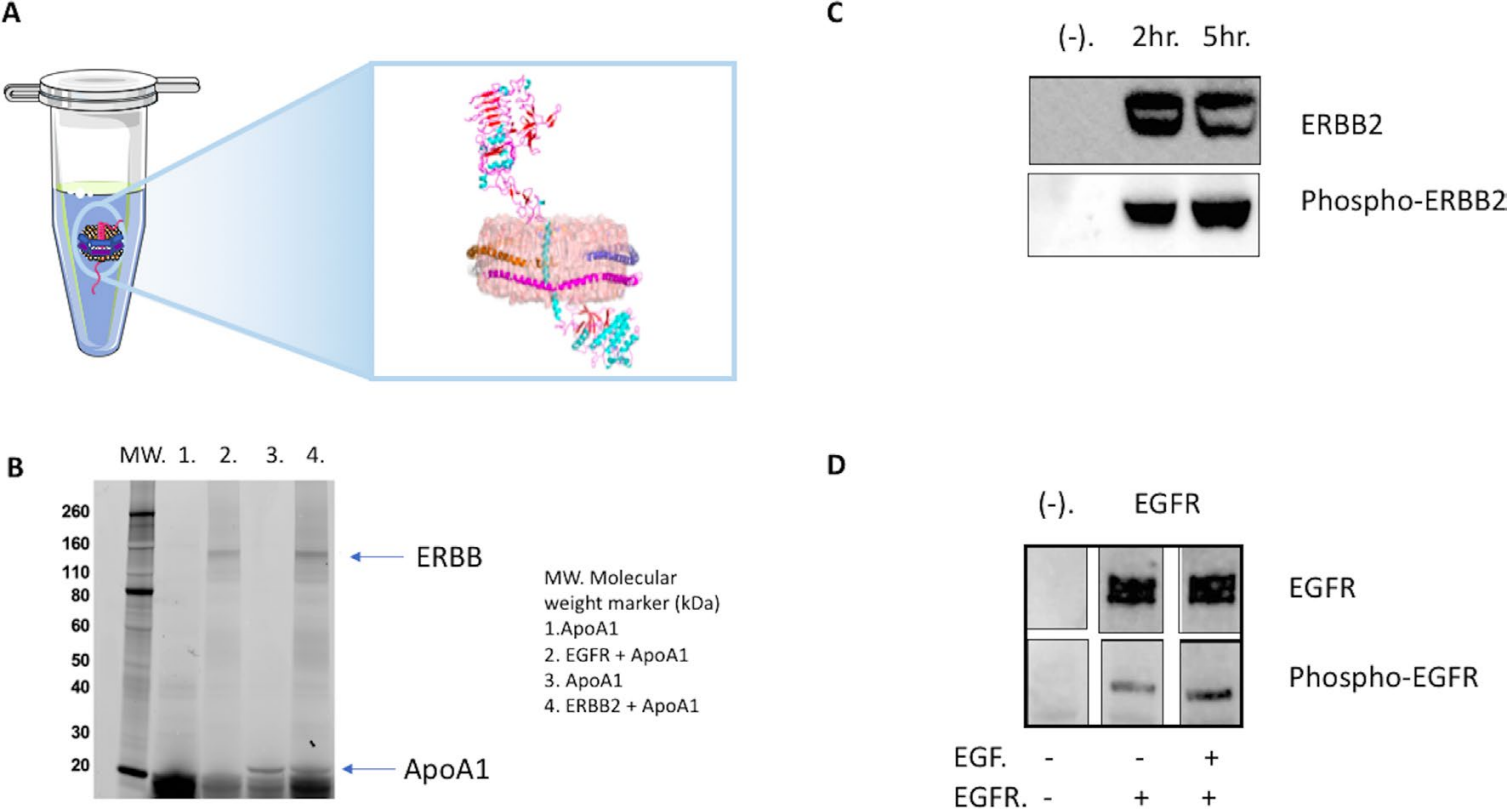
Cell-free co-expression for production of ERBB (erythroblastic oncogene B) tyrosine kinase complexes. **(A)** Illustrated ERBB2 receptor embedded in an NLP *via* cell-free expression and self-assembly. **(B)** Analysis of the co-expression of EGFR (epidermal growth factor receptor) and ERBB2 in the presence of the plasmid encoding an *ApoA1* gene *via* 4–20% sodium dodecyl sulfate polyacrylamide gel electrophoresis (SDS-PAGE). Visualization was accomplished *via* the incorporation of a fluorescent bodipy lysine dye. **(C)** NLP associated ERBB2 is tyrosine phosphorylated. Cell-free expressions were set up with and without (−) ERBB2 plasmid. Samples were collected at 2 h, and 5 h, resolved by SDS-PAGE and western blotted with anti-phospho-tyrosine ERBB2 antibody pY1248 and anti-ERBB2 antibody Ab-3 after stripping. **(D)** NLP associated EGFR is phosphorylated, and the presence of EGF in the cell-free reaction increases the level of phosphorylation. EGFR-NLPs showed low level of phosphorylation during cell-free expression. Adding EGF, the natural ligand of EGFR, increased the phosphorylation. Cell-free mixtures were resolved by SDS-PAGE and western blotted with anti-phospho-tyrosine EGFR antibody pY1110 and anti-EGFR. Images were spliced lanes from the same Western blot.

The combination of cell-free expression and NLP technology eliminates these issues and produces full length, tag-less and homogeneous ERBB receptors (**Figure 4**). The cell-free system lowers the possibility of co-purifying RTK-related contaminants and ensures the purity of the target protein, whereas NLPs provide the native lipid environment to maintain the correct ERBB folding. We have demonstrated that full-length ERBB2 and EGFR can be expressed in an *E. coli* lysate-based cell-free system (**Figure 4B**) (He et al., 2015; Quinn et al., 2018). Surprisingly, this is one of the first examples of producing functional TRKs in *E. coli*–derived lysates (He et al., 2015). Ni-NTA-based purification *via* a His tag on the NLP scaffold generated sufficient amount of full-length ERBB receptor for biochemical and biophysical characterization and structural analysis (He et al., 2015; Quinn et al., 2018).

Assays to study the function of the ERBB family of receptors are well-developed and include ligand binding and kinase activity assays (**Figure 4C and D**). *In vivo*, the binding of ERBB receptors to its ligand triggers dimerization, which in turn activates kinase activity and results in receptor phosphorylation or auto-phosphorylation. The process of auto-phosphorylation relies on dimerization to trigger signal transduction (Franklin et al., 2004; Badache and Goncalves, 2006). We have demonstrated that the cell-free preparation of full-length EGFR-NLP is capable of auto-phosphorylation at tyrosine residue 1110 with and without the binding of its ligand EGF (He et al., 2015). Phosphorylation is enhanced when the receptor binds to its natural ligand EGF, indicating that the kinase and the ligand binding domains are properly folded within the NLP. In addition, the proper response to ligand stimulation also suggests the receptor is capable of dimerization to induce auto-phosphorylation. Similar to EGFR, when co-expressed with NLPs the ERBB2 receptor is auto-phosphorylated at tyrosine residue 1248 (He et al., 2015). However, in the case of ERBB2, such phosphorylation is independent of ligand as there is no known ligand to ERBB2. In fact, the level of ERBB2 auto-phosphorylation appears coincident with the accumulation of the receptor itself within the NLP. It is worth noting that in cancer cells, the activation of ERBB2 is often associated with amplification and overexpression of ERBB2, in which the crowding of the receptor may be localized to specific regions of the cell (Badache and Goncalves, 2006; Moasser, 2007). Because no known ligand has been identified that binds to ERBB2 it is impossible to assess extracellular domain folding *via* a ligand binding assay. However, there are other ways to address this problem. The ERBB2 antibody trastuzumab is known to bind to the extracellular domain of ERBB2 only when it is correctly folded. We have demonstrated that cell-free preparations of dimerized ERBB2 bind trastuzumab with the same K_d_ as measured from mammalian cell ERBB2 preparations, indicating proper folding of the receptor extracellular domains (He et al., 2015). Collectively, these observations indicate that the cell-free expressed ERBB family proteins are correctly folded and functional when associated with the NLP.

In addition, the ERBB-NLP complex provides an ideal platform for structural studies of this family of receptors using small angle X-ray scattering (SAXS) (He et al., 2015) and single-molecule FÖrster resonance energy transfer (FRET) (Quinn et al., 2018). SAXS is a technique commonly used to assess the electron density envelope (and thus low-resolution conformation) of protein complexes and particles. The SAXS scattering profile for purified ERBB2-NLP not only confirmed the presence of ERBB2 insertion, but also provided low resolution structural information such as radius of gyration of the complex indicative of the conformational and oligomerization state of the receptor inserted in an NLP (He et al., 2015).

Furthermore, the highly flexible cell-free expression system enables straightforward engineering of the receptors with fluorescence and affinity tags to look at intermolecular folding events during auto-phosphorylation. These new capabilities afford us the opportunity to address unresolved mechanistic questions concerning the receptors’ structure-function relationships that have been intractable by other methods. For instance, by adding a fluorescent tag to the C-terminal protein, we observed through single-molecule FRET experiments that EGFR exists in multiple states with varying distance between the ATP binding pocket and the C-terminus of the protein (Quinn et al., 2018). Interestingly, FCS results also suggest compaction of the C-terminus of EGFR upon binding to ligand EGF. These results suggest a relationship between ligand association and a conformational change at the C-terminus of EGFR, a region that previously was considered to play no role in auto-phosphorylation and signal transduction. These initial results also pave the path towards investigation of more complicated transient and/or heterogeneous processes that may play critical functional roles in the ERBB family of receptors.

## Summary and Future Perspectives

Over the last decade, NLPs have been a major tool for addressing important questions associated with membrane proteins. Cell-free one-pot assembly of NLPs is a robust method for effective solubilization of membrane proteins without the need for detergent exchange and removal. Second, control over disc structure can be asserted through protein engineering and adjusting the ratio of protein to lipid during NLP formation. Cell-free methods no longer require pre-formation of NLPs and allow *de novo* production of membrane-bound proteins. Furthermore, large functional complexes can be simultaneously produced using the co-expression methods described. This review only highlights a small portion of the membrane proteins studied using the one-pot method, and it should be acknowledged that several other groups have extended this technique to produce a greater variety of membrane proteins (Malhotra and Alder, 2017; Dondapati et al., 2018).

The NLPs have provided a highly flexible platform for solubilizing functional membrane proteins, and their adaption continues to grow. However, it should be noted that some limitations exist for both the cell-free methods and for studying the NLP-inserted receptors. Although cell-free expression is a powerful method for membrane protein production, it has potentially low protein yield and high cost of production. However, steady progress toward improving the cell-free protein expression levels and reducing cost is being made. For example, the Germany-based company LenioBio GmbH has developed a plant-based cell-free system that can produces up to 3 mg/ml protein in a single ml reaction. Other research groups from United States have also optimized methods for cell-free lysate preparation that reduced the cost to US$0.021 per µg of protein produced making the cell-free technology a more promising method (Levine et al., 2019). It should also be noted that when a membrane protein is inserted into NLP, both sides of the protein are exposed to the aqueous solution. Although this is great for solution-based structural studies and ligand binding assays, it is very difficult to use the membrane protein-NLP complex for direct patch clamp or molecular pumping studies. One of the ways to accomplish such an experiment is to fuse the membrane protein NLPs to lipid bilayer. However, such fusion is not always efficient, and the mechanism of the fusion is not clear. Studies aiming to elucidate the key factors affecting the fusion of NLP to lipid bilayer is current ongoing in our laboratories. A second limitation lies in the compartmentalization of membrane proteins. The interaction between multiple membrane proteins across different NLPs maybe weakened by the confinement within an individual NLP. This would be especially true for interactions mediated by the transmembrane domain of the proteins. Another key limitation is a lack of control over the orientation of the membrane protein inserted. In theory, two membrane proteins can insert in different directions, aligning the intracellular part of one protein with the other’s extracellular component. Such a “dimer” may be biologically irrelevant and would potentially complicate any structural or functional studies. In addition, the membrane protein-NLP complex may be stable for days after it is formed, but the long-term stability of such complex in solution is still problematic. A possible way for long-term storage of NLP complex is through lyophilization; however, this needs further investigation.

The goal of this review is to highlight the impact of the single-step cell-free co-translation technology for expressing NLPs/nanodiscs to study GPCRs and RTKs. The early experiments focused on demonstrating that cell-free generated NLPs were equivalent to those formed from detergent-solubilized components, while enabling functional studies of membrane proteins. Of importance has been the finding that *E. coli* lysate-based preparations are compatible with the production of functional GPCRs and TRKs when co-translated, producing NLP complexes in the presence of lipids. Further, we have recently demonstrated that NLP-associated GPCRs can be transferred from *in vitro* to the cellular environment to fully recapitulate signal transduction, thus influencing cellular phenotype (Patriarchi et al., 2018).

The NLP-associated GPCRs are amenable to various methods of assessing activity and may be readily applied to high-throughput screening schemes for GPCR targeted drug discovery. For example, single-membrane proteins supported in NLPs have been screened against drug candidate libraries and their ligand binding assessed through localized surface plasmon resonance (LSPR), including cytochrome P450s (Das and Sligar, 2009) and human adenosine (A2A) GPCR (Bocquet et al., 2015). NLP-based screening strategies have recently been pursued that establish libraries of NLP encapsulated membrane proteins prepared from both bacterial (Marty et al., 2013; Roy et al., 2015) and mammalian membrane systems (Wilcox et al., 2015; Gregersen et al., 2016; Mak et al., 2017). Although these studies did not specifically screen drug candidates against GPCRs, some mammalian libraries have shown GPCR enrichment as well as tyrosine kinase activity (Gregersen et al., 2016). Alternatively, the association with NLPs may make GPCRs easily delivered and integrated into cellular membranes *in vitro* and possibly *in vivo*, facilitating a novel method of manipulating both cellular protein content and behavior.

The cell-free production of ERBB receptor tyrosine kinases has proven to be an effective tool to facilitate research of this important protein family. Prior to cell-free production, it was not possible to produce a homogeneous preparation of the full-length receptor in a soluble form for biochemical studies. This approach may also provide the possibility to better characterize post translation effects, heterodimerization with other receptors, and the impact of mutations on function for ERBB receptors. Given the demonstrated role of up-regulation in a number of cancers, facile production of functional ERBB receptor tyrosine kinases should prove useful for the design of novel cancer therapeutics that target various ERBB’s. For example, by producing ERBB mutants by cell-free expression for combinatorial cancer drug screening.

Co-translation of the NLP platform has also proven useful for functional characterization using multiple biochemical and biophysical techniques (Blanchette et al., 2009b; Gao et al., 2011; He et al., 2013; Lai et al., 2015; Cleveland et al., 2018). Atomic force microscopy (AFM), EM, and FCS have been critical in general to facilitate characterization of the nanoparticles and the functional attributes of the embedded membrane proteins. Of note has been FCS, which was used to study particle size, monodispersity, protein–protein interactions, and structural aspects of GPCRs and RTKs (Gao et al., 2012; Quinn et al., 2018). This has allowed us to ask questions about the dynamic conformation of the proteins within the disc. It should be possible to study complex interactions with multiple proteins and lipids using this same approach, thus providing a new level of understanding regarding the complex protein conformational landscape in a near-native environment.

Over the last 5 years, NLPs have been providing insight into membrane protein structures by enabling TEM, SAXS, NMR, and crystallography experiments in native lipid conditions (Viegas et al., 2016; Puthenveetil et al., 2017). The role co-translation has played in these studies depends on its ability to produce large amounts of homogenous particles to address the requirement of preparation purity and uniformity associated with these biophysical techniques. Advances in cryo-EM detectors are revolutionizing direct imaging and structural characterization of membrane proteins, and several recent breakthroughs in membrane protein structural biology using cryo-EM owe their success to NLP-based protein preparations (Frauenfeld et al., 2011; Akkaladevi et al., 2013; Efremov et al., 2017; Stam and Wilkens, 2017; Matthies et al., 2018). Of interest will be developing experimental paths forward for generating protein crystal structures of membrane proteins in NLPs. It should also be noted that X-ray crystallography is rapidly developing to include new serial crystallography experimental strategies at X-ray free electron lasers (XFELs) and synchrotrons requiring less material and smaller crystals, which can be combined with other imaging modalities (Ishchenko et al., 2018). Microcrystal-based serial experiments enabled by XFELs have already seen great success in generating structures for membrane proteins otherwise adverse to large single crystal growth, and improvements in sample delivery and experimental design have opened the door to LCP-grown crystals and those with strict environmental requirements (Hunter et al., 2014; Weierstall et al., 2014; Roedig et al., 2016).

At the same time, NLP-based protein preparations may lead to new research capabilities in other model membrane systems such as the supported lipid bilayer (SLB). The SLB is a lipid bilayer that has been reconstituted on a planar support. Although SLBs are popularly used to probe membrane organization and dynamics (Macháň and Hof, 2010; Kiessling et al., 2015) and protein–lipid interactions (Rossi and Chopineau, 2007; Smith, 2012), efforts to utilize them for studying membrane proteins have traditionally been hindered by challenges in protein production and insertion (Tiefenauer and Demarche, 2012). Herein lies the opportunity to utilize cell-free expression and NLPs. Cell-free, NLP-based co-translation methods could directly address obstacles in protein accessibility by presenting efficient routes for membrane protein production. This mode of preparation could also overcome barriers to protein insertion. After all, cell-free expression has been demonstrated as a viable method for introducing proteins into SLBs *in situ* (Chalmeau et al., 2011; Cleveland et al., 2018). Moreover, NLPs have been shown to transfer proteins spontaneously to biological (Patriarchi et al., 2018) and model membranes (Lai and Renthal, 2013).

NLP-mediated delivery of membrane proteins into SLBs is an exciting goal for future research because of the broad applicability of SLBs. Due to their two-dimensional structure, SLBs are well suited for surface-sensitive applications, such as biochips (Chadli et al., 2018) and biosensors (Rebaud et al., 2014). Further, they are highly amenable to analysis by quartz crystal microbalance (Meléndrez et al., 2018), AFM (Bronder et al., 2016), X-ray (Kuhl et al., 2009; Xu et al., 2018), and neutron (Lind et al., 2015) scattering. Although conventional SLBs are formed on hard substrates (e.g., glass), an SLB can alternatively be modified to incorporate a polymer layer (El-khouri et al., 2011; Watkins et al., 2011; Pace et al., 2015; Andersson and Köper, 2016) between the bilayer and underlying hard substrate. Polymer layers (i.e., cushions, spacers) are beneficial for preventing unwanted protein-substrate interactions and for modeling the presence of an extracellular matrix (Tanaka and Sackmann, 2005; El-khouri et al., 2011). Overall, the SLB is a robust platform with substantial potential for exploring a vast array of membrane phenomena. Cell-free expression technologies and NLPs would unlock that potential by providing reliable access to a diverse selection of membrane proteins, and by presenting a mechanism for introducing them into SLBs while preserving structural fidelity and functionality.

Beyond engineering membrane proteins and demonstrating the utility of SLBs, the next major technological hurdle for cell-free membrane protein production is controlling post-translational modification of proteins to include eukaryotic receptors that require glycosylation to regulate function. Currently, a number of eukaryotic cell-free lysates, including those based on insect (Tarui et al., 2000; Tarui et al., 2001; Suzuki et al., 2007), rabbit reticulocytes (Hancock, 1995), and Chinese hamster ovary cells (Brödel et al., 2014), have been shown to be capable of glycosylation, but these are either low-yielding or the process is not well controlled. In recent years, progress has been made towards engineering *E. coli* strains to express glycol-regulating proteins for cell-free lysate production (Martin et al., 2017; Jaroentomeechai et al., 2018; Kightlinger et al., 2018; Schoborg et al., 2018) and some promise may lie in engineering mammalian-based systems to increase yield. In the future, co-translation can also be combined with some of the developing techniques in membrane protein solubilization, including amphipols, SMALPS and synthetic lipids to further characterize membrane-bound receptors, which mediate the spread of many cancers and are targets of multiple drugs as well as chemotherapeutics. The ability to study these receptors outside the cell environment will enable structural and mechanistic studies for improved understanding of carcinogenesis and other diseases. Given that membrane-associated proteins account for the majority of therapeutic drug targets, it is important to continue developing novel technologies to gain access to these important classes of proteins.

## Author Contributions

MS and WH contributed equally to this manuscript. The concepts and conclusions reviewed were generated jointly by MS, WH, AD, TK, and MC. MS, WH, AD, TK, and MC participated in the writing and editing of this manuscript.

## Funding

This work was performed under the auspices of the U.S. Department of Energy by Lawrence Livermore National Laboratory under Contract DE-AC52-07NA27344. Research was supported by the National Institutes of Health under award numbers R21AI120925, R01CA155642, and R01GM117342 and the National Science Foundation Chemistry division through grant CHE-1413745.

## Conflict of Interest Statement

The authors declare that the research was conducted in the absence of any commercial or financial relationships that could be construed as a potential conflict of interest.

## Abbreviations

NLPs, nanolipoprotein particles; GPCR, G-protein-coupled receptors; RTKs, tyrosine kinases; FCS, fluorescent correlation spectroscopy; EM, electron microscopy; AFM, atomic force microscopy.
